# *Phytophthora* diversity in urban forested streams of Fairfax County, Virginia, with *Phytophthora matreminventam* sp. nov. as the maternal parent of *P*. ×*stagnum*

**DOI:** 10.1371/journal.pone.0359718

**Published:** 2026-10-27

**Authors:** Devin Bily, Xiao Yang, Tashi Gyatso, Katherine Layton

**Affiliations:** 1 Department of Plant Pathology, Virginia Department of Agriculture and Consumer Services, Richmond, Virginia, United States of America; 2 Department of Plant Industry, Plant and Pest Diagnostic Clinic, Clemson University, Pendleton, South Carolina, United States of America; 3 Fairfax County Urban & Community Forestry Division, Fairfax, Virginia, United States of America; Franklin & Marshall College, UNITED STATES OF AMERICA

## Abstract

*Phytophthora* diversity in urban forested freshwater systems of Fairfax County, Virginia was investigated by sampling 15 streams in the spring and fall from 2021 to 2024. A total of 163 isolates were identified. Based on multilocus sequence and phylogenetic analyses, they were categorized into 14 taxa belonging to 7 *Phytophthora* subclades including *P. pseudocitrophthora* in subclade 2a, *P. pini* in subclade 2c, *P*. *chlamydospora*, *P. gonapodyides*, *P. matreminventam* sp. nov., *P.* taxon bitahaiensis, and *P.* t. sylvatica in subclade 6b, *P. lacustris* in subclade 6d, *P. honggalleglyana*, *P. irrigata*, *P. virginiana* sensu lato, and *P.* sp. × Hennops in subclade 9a, *P.* t. polonicoides in subclade 9b, and *P. intercalaris* in subclade 10c. Notably, *P. matreminventam* sp. nov. named in this study was determined to be the maternal parent of *P.* × *stagnum* based on their unique genetic traits. Although a few species recovered in this study reportedly possess innate pathogenicity to woody plants, no significant symptoms of *Phytophthora* diseases were observed in the nearby forests at the sampling sites.

## Introduction

*Phytophthora* de Bary is a diverse genus of phytopathogenic and saprobic oomycete microorganisms with 223 currently known taxa worldwide [1]. *Phytophthora* species are ubiquitous residents of freshwater streams in agricultural, forested, and urban landscapes, yet their aquatic ecology is poorly understood [2–10]. Most taxa recovered from aquatic ecosystems, especially in *Phytophthora* clades 6 and 9, are high temperature-tolerant facultative parasites that colonize leaf debris and do not cause significant harm to riparian plants [1,2,11,12]. A few aquatic species are notable pathogens of trees, including *P*. × *alni*, which has caused large-scale dieback on riparian alder (*Alnus* spp.) trees in Europe, and *P. lateralis* causing mortality of Port-Orford-cedar (*Chamaecyparis lawsoniana*) in California and Oregon [13]. Temperature and precipitation are the main drivers on species diversity, along with water chemistry attributes like nitrogen abundance and pH, and the highest quantity of zoospores are generally detected in spring and fall [2,11,12,14].

Repeated stream sampling can provide a representative panorama into the diversity of both terrestrial and aquatic *Phytophthora* taxa present within a watershed [3,4,8,9,11,15–17]. *Phytophthora* taxa recovered from aquatic ecosystems are rarely found in terrestrial ecosystems, yet terrestrial species can be found in aquatic environments from being washed out from soil in catchment areas [11,12]. There are exceptions, however, and aquatic clade 6 species including *P. chlamydospora*, *P. gonapodyides*, *P. inundata*, and *P. thermophila* have been identified from riparian soils and plants [15,18]. Urban or agricultural areas generally have significantly higher levels of species richness than natural environments and can act as a link for harmful species to invade natural ecosystems [4,12,15,19,20]. For example, *P. ramorum*, the causal agent of sudden oak death in the western United States, was introduced into the United States via the nursery trade and subsequently has killed millions of forest trees [21]. Therefore, urban waterways are ideal for monitoring *Phytophthora* taxa possibly transplanted from nurseries or landscaping.

In addition to a high species richness, aquatic environments are particularly favorable for interspecific hybridization due to facilitation in sexual interactions and ample nutrients [4,22–24]. *Phytophthora* species hybridize by inheriting and recombining alleles or genes from both parents, which can increase virulence or host range or facilitate adaptations to extreme environmental conditions [22–24]. Examples of hybrids include clade 1 species *P*. × *pelgrandis* and *P*. × *serendipita* from Peru and Taiwan, and clade 7 species *P.* × *alni* from Europe (Hurtado- [13,25]*.* Clade 6 is especially rich in hybrid taxa, including *P*. *lacustris* × *P. riparia* and *P*. × *stagnum* from the United States, and at least 12 hybrid species as a result of different parental combinations of *P*. *amnicola*, *P*. *fluvialis*, *P*. *litoralis*, *P*. *moyootj* and *P*. *thermophila* in South Africa and Australia [4,24,26,27]. Overall, interspecific hybridization and polyploidy are two processes that play an important role in the evolution of *Phytophthora* [26,28].

Fairfax County is the largest suburban ring of the Washington metropolitan area and the most populous county in Virginia [29]. Despite its dense population, forests and neighborhood trees cover 55% of its land area [30]. The most abundant tree species include red maple (*Acer rubrum*), tulip tree (*Lirodendron tulipifera*), American beech (*Fagus grandifolia*), and white oak (*Quercus alba*) [30]. This fluctuating landscape provides a unique scenario for understanding *Phytophthora* populations in waterways impacted by urban development. The objective of this survey was to characterize the *Phytophthora* taxa present in waterways of Fairfax County and detect exotic species that have the potential to be harmful to plant health.

## Materials and methods

### Stream sampling, culturing, and DNA extraction

Stream baiting was performed from 2021 to 2024 during spring and fall from April to May and September to November. In total, 15 streams were sampled and baited with asymptomatic *Rhododendron maximum* leaves following the ‘bottle of bait’ method from the *P. ramorum* Domestic Regulatory Program Manual [31] (Fig 1). Each sampling site was located on public land and no permits were required. One liter of stream water was collected in 100-ml aliquots from each sampling site. The leaf and leaf pieces were placed in the bottle after collection and incubated at 20 ± 2 °C. After 72 h, the leaves and leaf pieces were washed clean with distilled water, wrapped in a moistened paper towel, and shipped overnight to the laboratory in a sealed plastic bag. Upon receipt, the leaves were incubated at 20 ± 2 °C for up to an additional seven days, where 6-mm necrotic lesions from 10 leaf tissues were cut out with a hole puncher, surfaced disinfected with 70% ethanol for 1 min, and plated on CMA-PARP medium [corn meal agar (CMA) amended with pimaricin (10 mg/L), ampicillin (200 mg/L), rifampicin (10 mg/L), and pentachloronitrobenzene (5 mg/L) [32]] at 20 ± 2 °C. After three days, colonies were subcultured to V8 juice agar (V8A) and incubated at 20 ± 2 °C. After five days, mycelia were swabbed with a sterile needle, macerated in CD1 buffer with a BioSpec MiniBeadbeater-16 (Bartlesville, Oklahoma), followed by DNA extraction using the Qiagen DNeasy PowerLyzer Microbial Kit (Germantown, Maryland).

**Fig 1:**
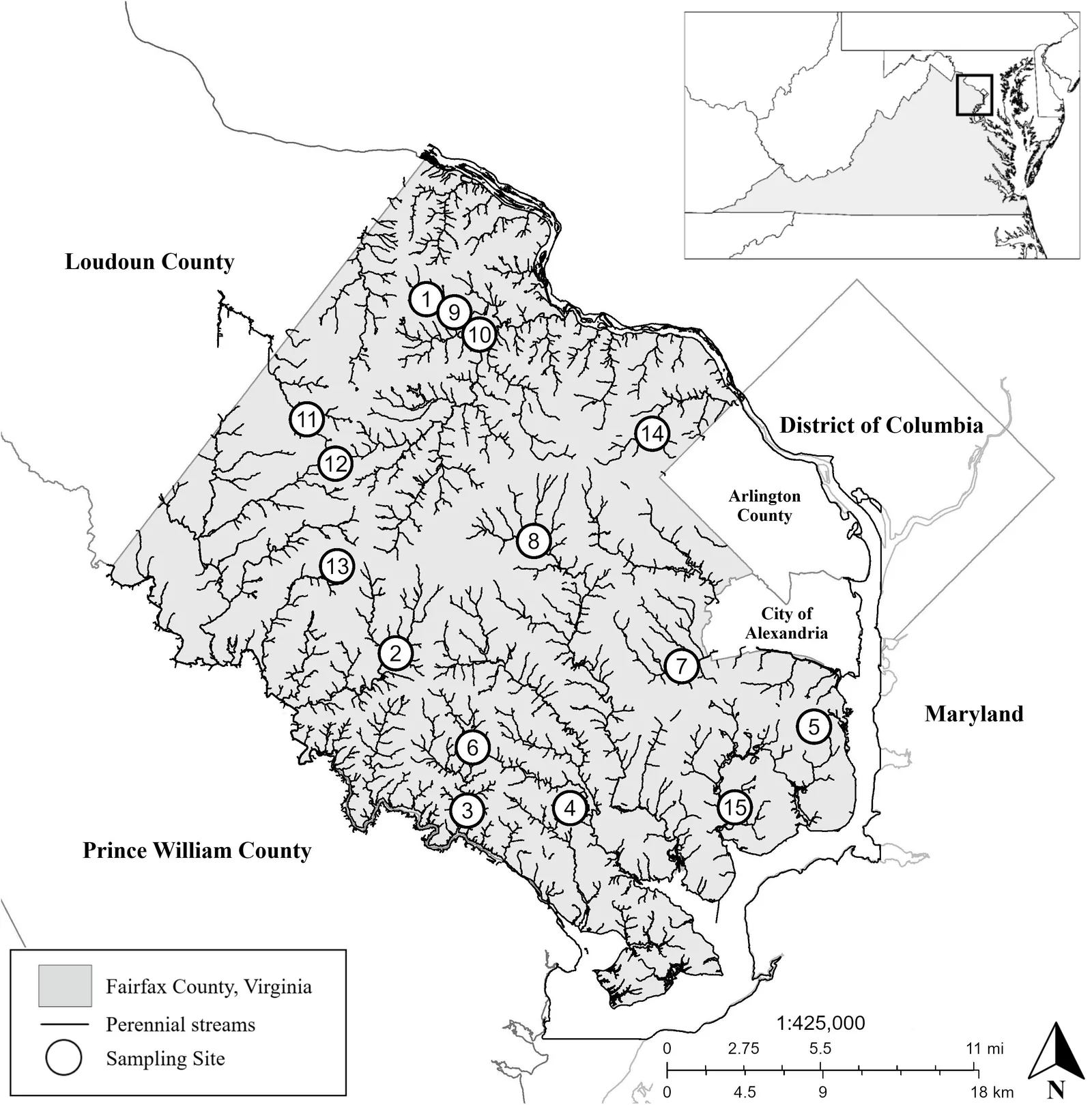
A map of the 15 streams sampled in Fairfax County, Virginia. Map image is the intellectual property of Esri and is used herein under license. Copyright © 2026 Esri and its licensors. All rights reserved.

### PCR and sequencing

Conventional PCR was performed with a Biometra T3000 thermocycler (Gottingen, Germany), where each DNA template was run undiluted and at a 1:10 dilution with molecular biology grade water. Gel electrophoresis was performed on a ThermoScientific Owl A1 gel system (Waltham, Massachusetts) with 1% Invitrogen Ultrapure agarose (Carlsbad, California) and an Invitrogen TrackIT 100-bp ladder (Carlsbad, California) in Tris-acetate-EDTA buffer for 90 mins at 100V. The gels were stained in ethidium bromide (50µl/L) for 15 mins while oscillating before being washed and visualized with an Axygen gel documentation system (Corning, New York).

For DNA barcoding, the nuc rDNA ITS1-5.8S-ITS2 internal transcribed spacer (ITS) region and the cytochrome *c* oxidase subunit 1 (*cox1*) gene were sequenced for all isolates using the primer sets ITS5/ITS4 [33] and OomCoxI-Levup/Fm85mod [34], respectively. Additionally, the β-tubulin (*βtub*) gene and/or the heat shock protein 90 (*hsp90*) gene were sequenced to accurately identify taxa that cannot be resolved by ITS and *cox1* sequences using the primer sets TUBUF2/TUBUR1 [35] and HSP90_F1/HSP90_R1 [36], respectively. PCR amplicons were purified using ExoSAP-IT PCR Product Cleanup Reagent (Affymetrix, Santa Clara, California) and Sanger sequenced at Eurofins Genomics (Louisville, Kentucky). Bidirectional nucleotide sequences for each isolate were trimmed and aligned using GENEIOUS 2023.21 software (Biomatters, Auckland, New Zealand). Consensus sequences were deposited to GenBank and used to identify the isolates by comparing them to type and authenticated isolates of the respective taxa (Table 1).

**Table 1:** The representative isolates, collection data, and NCBI GenBank accession numbers generated from the study and the reference specimens used for molecular analyses.

Subclade	Species	Isolate^a^	Other collection number	Area of Collection	GenBank accession number^b^				Reference
					ITS	*cox1*	*βtub*	*hsp90*	
2a	*P. citrophthora*	CBS 950.87^T^	ATCC 52231; CPHST BL 60	California, USA	**HQ643205**	**OK574289**	**MT583638**	–	[37]
	*P. pseudocitrophthora*	CBS 149500^T^		Sicily, Italy	**OR518931**	**OR526136**	**OR558053**	–	Jung et al. [38]
		21FFX-014B		Fairfax County, Virginia, USA	**PZ168792**	**PZ170349**	**PZ170367**	–	This study
2c	*P. pini*	CBS 181.25^T^	ATCC 64532; CPHST BL 48	Minnesota, USA	**KF317079**	**KF317100**	**KX250811**	–	Hong et al. [39]
		21FFX-022		Fairfax County, Virginia, USA	**PZ168789**	**PZ170346**	**PZ170364**	–	This study
		23FFX-015A		Fairfax County, Virginia, USA	**PZ168790**	**PZ170347**	**PZ170365**	–	This study
		24FFX-009D		Fairfax County, Virginia, USA	**PZ168791**	**PZ170348**	**PZ170366**	–	This study
6b	*P. borealis*	CBS 132023^T^	ATCC MYA-4881; WPC P19794; AKWA58.1-0708; CPHST BL 93; 60B2	Alaska, USA	**HM004232**	**MH136854**	–	–	Hansen et al. [37,40]
	*P. chlamydospora*	CBS 149403^T^	WPC P6133; CPHST BL 156	UK	**MG865471**	**MH136867**	–	–	Hansen et al. [37,41]
		21FFX-017		Fairfax County, Virginia, USA	**PZ168774**	**PZ170331**	–	–	This study
	*P. crassamura*	CBS 140357^T^	PH138; CPHST BL 150A	Italy	**MG865481**	**MK493474**	–	–	Scanu et al. [37,42]
	*P. gonapodyides*	CPHST BL 172^T^	WPC P6992	USA	**MG865502**	**MH136897**	–	–	[37]
		24FFX-002B		Fairfax County, Virginia, USA	**PZ168776**	**PZ170333**	–	–	This study
		24FFX-015A.1		Fairfax County, Virginia, USA	**PZ168775**	**PZ170332**	–	–	This study
	***P. matreminventam* sp. nov.**	22FFX-029B^T^		Fairfax County, Virginia, USA	**PZ168804**	**PZ170361**	**PZ170368**	**PZ170371**	This study
		21FFX-026		Fairfax County, Virginia, USA	**PZ168805**	**PZ170362**	**PZ170369**	**PZ170372**	This study
	*P. megasperma*	CBS 402.72^T^	ATCC 58817; IMI 32035; WPC P6957; CPHST BL 43; 62C7	Washington DC, USA	**MG865535**	**MH136930**	–	–	[37]
	*P. mississippiae*	ATCC MYA-4946^T^	57J3; WPC P19994; CPHST BL 126	Mississippi, USA	**KF112852**	**MH136935**	–	–	Yang et al. [37,43]
	*P.* × *stagnum*	ATCC MYA-4926^T^	43F3	Virginia, USA	**KJ705084** ^**c**^	**LC595955**	**KJ883155**	**PZ366123**	Yang et al. [24]
	*P.* taxon bitahaiensis	DQ09−5		China	**JQ755230**	**KT899394**	–	–	Huai et al. [44]
		21FFX-019		Fairfax County, Virginia, USA	**PZ168772**	**PZ170329**	–	–	This study
		21FFX-024		Fairfax County, Virginia, USA	**PZ168773**	**PZ170330**	–	–	This study
	*P.* taxon sylvatica	VN896		Viet Nam	**MN872782**	**MN866104**	–	–	Jung et al. [45]
		23FFX-022A		Fairfax County, Virginia, USA	**PZ168793**	**PZ170350**	–	–	This study
		24FFX-022B		Fairfax County, Virginia, USA	**PZ168794**	**PZ170351**	–	–	This study
6d	*P. lacustris*	P245^T^	WPC P10337; CPHST BL 69	Bexley Heath, Kent, UK	**AF266793**	**JF896561**	–	–	Nechwatal et al. [46]
		22FFX-006		Fairfax County, Virginia, USA	**PZ168787**	**PZ170344**	–	–	This study
		22FFX-015A.2		Fairfax County, Virginia, USA	**PZ168788**	**PZ170345**	–	–	This study
9a	*P. honggalleglyana*	ATCC MYA-4460^T^	5D1; WPC P19616; CPHST BL 68	Virginia, USA	**EU583793**	**KC733452**	–	–	Hong et al. [47]
		21FFX-011A		Fairfax County, Virginia, USA	**PZ168781**	**PZ170338**	–	–	This study
		24FFX-024B		Fairfax County, Virginia, USA	**PZ168782**	**PZ170339**	–	–	This study
	*P. irrigata*	ATCC MYA-4457^T^	WPC P16861; CPHST BL41	Virginia, USA	**EU334634**	**KC733453**	–	–	Hong et al. [48]
		22FFX-009		Fairfax County, Virginia, USA	**PZ168786**	**PZ170343**	–	–	This study
		24FFX-029B		Fairfax County, Virginia, USA	**PZ168785**	**PZ170342**	–	–	This study
	*P. virginiana* sensu lato	ATCC MYA-4927^T^	46A2; WPC P19829; CPHST BL 165	Virginia, USA	**KC295544**	**MH477761**	–	–	Yang & Hong [43]
		21FFX-014C		Fairfax County, Virginia, USA	**PZ168795**	**PZ170352**	–	–	This study
		21FFX-023		Fairfax County, Virginia, USA	**PZ168796**	**PZ170353**	–	–	This study
		22FFX-005A		Fairfax County, Virginia, USA	**PZ168799**	**PZ170356**	–	–	This study
		22FFX-019		Fairfax County, Virginia, USA	**PZ168797**	**PZ170354**	–	–	This study
		22FFX-028B		Fairfax County, Virginia, USA	**PZ168798**	**PZ170355**	–	–	This study
		23FFX-008		Fairfax County, Virginia, USA	**PZ168801**	**PZ170358**	–	–	This study
		23FFX-019B		Fairfax County, Virginia, USA	**PZ168800**	**PZ170357**	–	–	This study
		24FFX-022C		Fairfax County, Virginia, USA	**PZ168803**	**PZ170360**	–	–	This study
		24FFX-030A		Fairfax County, Virginia, USA	**PZ168802**	**PZ170359**	–	–	This study
	*P*. sp. × Hennops	CMW 33363		South Africa	**GU799640**	**GU799663**	–	–	Oh et al. [49]
		21FFX-011B		Fairfax County, Virginia, USA	**PZ168779**	**PZ170336**	–	–	This study
		21FFX-016B		Fairfax County, Virginia, USA	**PZ168778**	**PZ170335**	–	–	This study
		24FFX-027B		Fairfax County, Virginia, USA	**PZ168777**	**PZ170334**	–	–	This study
		24FFX-028A		Fairfax County, Virginia, USA	**PZ168780**	**PZ170337**	–	–	This study
9b	*P. polonica*	CBS 119650^T^	UASWS0198; WPC P19522; CPHST BL 109	Kolo, Poland	**DQ396410**	**MH136960**	**DQ399844**	**MK020373**	Belbahri et al. [50]
	*P. pseudopolonica*	CBS 142610^T^	LS06.3.2; CPHST BL 221	China	**KY707115**	–	–	–	Li et al. [51]
	*P.* taxon polonicoides	22FFX-029A		Fairfax County, Virginia, USA	**PZ168806**	**PZ170363**	**PZ170370**	**PZ170373**	This study
10c	*P. intercalaris*	CBS 140632^T^	45B7	Rapidan Stream, Virginia, USA	**KT163268**	**KT163315**	–	–	Yang et al. [10]
		22FFX-003		Fairfax County, Virginia, USA	**PZ168784**	**PZ170341**	–	–	This study
		24FFX-008B		Fairfax County, Virginia, USA	**PZ168783**	**PZ170340**	–	–	This study
	*P. scadinavica*	CBS 149204^T^	SW325	Sweden	**ON000786**	**ON013852**	–	–	Jung et al. [52]
1 (outgroup)	*P. nicotianae*	CPHST BL 44		Indonesia	**MG865550**	**MH136943**	–	–	[37]

^a^A superscript “T” designates the type isolate. ATCC: American Type Culture Collection, Manassas, Virginia, USA; CPHST: Center for Plant Health Science and Technology, USDA-APHIS PPQ, Beltsville, Maryland, USA; CMW: Collection of the Forestry and Agricultural Biotechnology Institute (FABI), University of Pretoria, Pretoria, South Africa; CBS: Westerdijk Fungal Biodiversity Institute, Utrecht, Netherlands; WPC: World Phytophthora Collection, University of California, Riverside, USA. ^b^ITS: nuclear ribosomal internal transcribed spacer 1, 5.8S spacer region ribosomal RNA, internal transcribed spacer 2 region; *cox1*: cytochrome *c* oxidase subunit 1; *βtub*: beta-tubulin; *hsp90*: heat-shock protein 90. ^c^The ITS sequence of *P.* × *stagnum* ex-type ATCC MYA-4926 contains profuse ambiguous polymorphic sites due to its hybrid nature and is unreadable. One of its cloned ITS sequence types (816-bp-type-1) is used for molecular analyses in this study.

### Phylogenetic analysis

ITS and *cox1* sequences of representative isolates obtained in this study and reference isolates listed in Table 1 were aligned individually using MUSCLE 5.1, then concatenated in MEGA 11 [53]. A maximum likelihood phylogeny was reconstructed using the concatenated sequences in RAXMLGUI 2.0 [54] with the general time-reversible substitution matrix plus gamma substitution rates and a proportion of invariable (GTR+GAMMA+I) and 1,000 bootstrap replicates. *Phytophthora nicotianae* isolate BL44 was used as the outgroup.

### Morphology and cardinal temperatures

Colony characteristics of a provisionally new species were described from isolate 22FFX-29B on V8A, CMA, CMA-PARP, malt extract agar (MEA), and potato dextrose agar (PDA) after 7 days at 25 °C with ambient light. Sporangia were produced by placing an agar plug taken from the edge of a 7-day-old isolate and flooding it in spring water for 24 h at 20 ± 2 °C under indirect light. Sporangia were measured using an Olympus BX43 microscope (Center Valley, Pennsylvania) at 1000 × magnification.

For temperature-growth relationships, a 5-mm diameter disc from isolates 21FFX-26 and 22FFX-29B was transferred onto 90-mm-diam V8A plates (three replicates each) and allowed to grow at 25ºC. After 24 h, the edge of the colony was marked before placing the plates upside down in 2.5, 5, 10, 15, 20, 25, 30, 35, and 37.5 ºC incubators. After 7 d, radial growth of the colonies was measured along four, right-angled lines that intersect the center of the colony and the mean growth rates were calculated. Plates that showed no growth at 2.5 and 37.5 ºC were returned to 25 ºC to determine lethal temperatures.

## Results

### Stream survey

A total of 179 *Phytophthora* isolates were recovered from 15 streams during the spring and fall of 2021–2024 (Table 2). The streams that were sampled the most recovered the highest quantity of isolates and species diversity (Table 2). The stream with the most species was Pimmit Run – McLean (*n* = 9), followed by Horsepen Run – Herndon (*n* = 7), North Fork Dogue Creek – Alexandria (*n* = 7), and Piney Run – Great Falls (*n* = 7). The most abundant species was *P. chlamydospora*, which was recovered in all months, followed by *P. honggalleglyana* (previously *P. hydropathica*), *P. virginiana* sensu lato, *P. irrigata*, and a previously unknown species in subclade 6b that is officially named *as P. matreminventam* sp. nov. in the section below (Table 3). These five species comprised 78% of the total isolates. Over the four-year period, the month with the highest isolates recovered was November (*n* = 60), followed by October (*n* = 55), May (*n* = 42), June (*n* = 10), April (*n* = 8), and December (*n* = 4) (Table 3).

**Table 2:** The number of *Phytophthora* isolates and taxa recovered from each stream and year.

No.	Sample Site			Number of samples collected by year				Isolates recovered				Total No. *Phytophthora* taxa
		Latitude	Longitude	2021	2022	2023	2024	2021	2022	2023	2024	
**1**	Piney Run - Reston	38.985556	-77.327231	0	2	2	1	n/a	2	2	4	4
**2**	Popes Head Creek - Fairfax Station	38.802162	-77.347635	0	2	2	3	n/a	4	4	8	5
**3**	Sandy Run - Fairfax Station	38.720653	-77.299969	0	2	1	1	n/a	2	2	2	4
**4**	Rocky Branch - Lorton	38.721931	-77.231624	0	2	1	1	n/a	2	3	2	4
**5**	Paul Spring Branch - Alexandria	38.764129	-77.069299	0	2	1	1	n/a	2	3	1	3
**6**	South Run - Fairfax Station	38.753354	-77.296355	0	1	2	1	n/a	3	3	2	6
**7**	Backlick Run - Alexandria	38.795495	-77.157306	0	2	1	2	n/a	4	2	6	5
**8**	Bear Branch, Accotink Creek - Fairfax	38.860426	-77.255758	0	1	1	2	n/a	1	2	2	5
**9**	Piney Run - Great Falls	38.978756	-77.308584	0	2	2	2	n/a	3	3	7	7
**10**	Colvin Run - Vienna	38.967221	-77.292004	0	2	2	2	n/a	3	3	5	6
**11**	Horsepen Run - Herndon	38.923453	-77.406685	5	3	0	1	8	7	n/a	4	7
**12**	Cub Run - Herndon	38.900404	-77.387738	2	1	3	2	2	4	5	5	5
**13**	Little Rocky Run - Fairfax	38.847359	-77.386637	4	3	1	1	6	3	3	3	5
**14**	Pimmit Run - McLean	38.916017	-77.177012	3	2	2	2	5	3	4	6	9
**15**	North Fork Dogue Creek - Alexandria	38.722309	-77.122088	4	2	2	2	4	6	4	5	7

**Table 3:** The *Phytophthora* species identified by month of year over the four-year sampling period.

*Phytophthora* taxon	Month						Total
	Apr	May	Jun	Oct	Nov	Dec	
*P. chlamydospora*	6	17	5	10	18	1	57
*P. honggalleglyana*	0	6	1	15	4	0	26
*P. virginiana* sensu lato	0	1	0	11	7	0	19
*P. irrigata*	0	2	1	4	6	0	13
*P. matreminventam* sp. nov.	0	2	0	1	7	2	12
*P.* t. bitahaiensis	0	1	0	5	4	1	11
*P.* sp. × Hennops	0	0	1	3	2	0	6
*P. pini*	0	3	1	0	1	0	5
*P. intercalaris*	0	4	0	0	0	0	4
*P. gonapodyides*	2	1	0	0	0	0	3
*P. lacustris*	0	2	0	1	0	0	3
*P.* t. sylvatica	0	0	0	1	1	0	2
*P. pseudocitrophthora*	0	0	0	1	0	0	1
*P.* t. polonicoides	0	0	0	0	1	0	1
Unidentified isolates	0	3	1	3	9	0	16
Total	8	42	10	55	60	4	179

### Molecular identification

All 179 isolates obtained from the survey were processed for ITS and *cox1* DNA barcoding, resulting in informative sequences for a total of 163 isolates. They were identified as 14 taxa belonging to 7 *Phytophthora* subclades. Most isolates were identified based on 99–100% sequence identity levels to that of type or authenticated isolate of the respective species in each genetic marker. In a phylogenetic tree based on concatenated ITS and *cox1* sequences, all representative isolates obtained in the survey clustered with reference isolates of their respective taxa and closely related species (Fig 2). Exceptionally, the ITS sequence (GenBank accession no. PZ168806) of isolate 22FFX-029A is < 98% identical to that of any known species and its *cox1* sequence (PZ170363) is 99% identical (735/742 nt.) to that of the ex-type isolate of *P.* *polonica* (MH136960). It hereby is informally designated as a provisional taxon “*P.* t. polonicoides”. *βtub* and *hsp90* sequences were also generated for isolate 22FFX-029A (Table 1).

**Fig 2:**
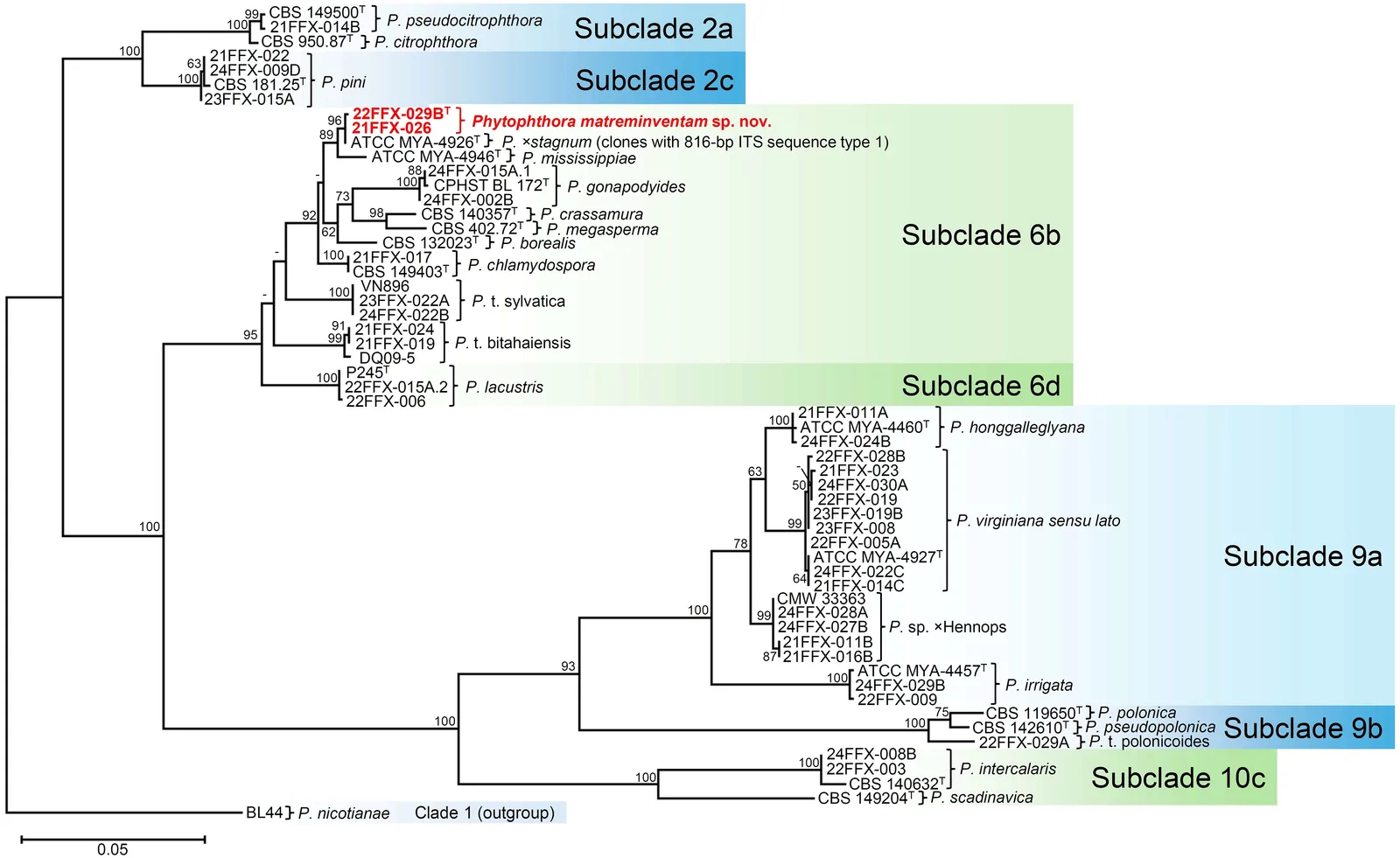
A maximum likelihood phylogenetic tree of *Phytophthora* taxa identified in this study based on concatenated sequences of the internal transcribed spacer region and the cytochrome c oxidase subunit 1 gene. Bootstrap support values (%) are given near nodes and those <50% are labeled as “-”. A superscript “T” designates the type isolate. Isolates of *Phytophthora matreminventam* sp. nov. officially named in this study are highlighted in red.

For isolates in *Phytophthora* clade 2 that could not be resolved with ITS and *cox1* sequences, the representative isolates including 21FFX-014B, 21FFX-022, 23FFX-015A, and 24FFX-009D were further identified based on their *βtub* sequences. The *βtub* sequence of isolate 21FFX-014B (PZ170367) was 99.8% identical (865/867 nt.) to that of the ex-type isolate of *P. pseudocitrophthora* (OR558053) and was 99.2% identical (860/867 nt.) to that of the ex-type isolate of *P. citrophthora* (MT583638). The other three isolates produced *βtub* sequences (Table 1) that were 100% identical to that of the ex-type isolate of *P. pini* (KX250811).

### Molecular characterization of *P. matreminventam* sp. nov

Isolates of *P. matreminventam* sp. nov. possess molecular characteristics of the maternal parent of *P. × stagnum*. First, the maternally inherited *cox1* sequences of representative isolates 22FFX-029B (PZ170361) and 21FFX-026 (PZ170362) are 100% identical to that of the ex-type isolate ATCC MYA-4926 of *P. × stagnum* (LC595955). Second, the ITS sequences of isolates 22FFX-029B (PZ168804) and 21FFX-026 (PZ168805) are 100% identical to one of the cloned ITS sequence types of isolate ATCC MYA-4926 (KJ705084). Third, the 21 dimorphic nucleotide sites in the *βtub* sequence of *P. × stagnum* ex-type isolate contain the monomorphic sequences of both *P. chlamydospora* ex-type P236 and isolate 22FFX-029B (Table 4). Similarly, the 11 dimorphic *hsp90* sequence sites of isolate ATCC MYA-4926 contain the monomorphic sequences of both *P. chlamydospora* ex-type and isolate 22FFX-029B (Table 4).

**Table 4:** Single nucleotide polymorphic sites found in the beta-tubulin (*βtub*) and heat shock protein 90 (*hsp90*) gene sequence alignments of *Phytophthora ×stagnum*, its paternal parent *P. chlamydospora*, and its material parent *P. matreminventam* sp. nov. Site numbers are given based on the alignments generated using CLUSTAL W in MEGA 11. Shading color: green indicates dimorphic sites; yellow indicates sequences characteristic of the paternal parent; blue indicates sequences characteristic of the maternal parent. ^a^A superscript “T” designates the type isolate. ^b^Y = T and C; S = G and C; M = A and C; R = G and A.

Species	Isolate^a^	GenBank Accession No.	Site number in aligned *βtub* sequences^b^																							
			261	276	306	327	342	354	429	450	462	519	561	571	594	621	634	667	702	723	726	759	783	789	993	1098
*P*. *chlamydospora*	P236^T^	KF750602	C	T	G	C	A	C	T	C	A	G	G	T	C	T	T	C	T	G	C	C	T	G	T	T
*P*. ×*stagnum*	ATCC MYA-4926^T^	KJ883155	Y	Y	S	C	M	Y	Y	Y	R	R	S	Y	M	Y	Y	Y	Y	G	Y	M	Y	G	Y	Y
*P. matreminventam* sp. nov.	22FFX-029B^T^	PZ170368	T	C	C	Y	C	T	C	T	G	A	C	C	A	C	C	T	C	K	T	A	C	R	C	C
Species	Isolate^a^	GenBank Accession No.	Site number in aligned *hsp90* sequences^b^																							
			204	285	330	342	387	483	639	693	789	792	801	870	873											
*P*. *chlamydospora*	P236^T^	MK020285	A	C	T	C	C	T	A	C	T	T	A	C	G											
*P*. ×*stagnum*	ATCC MYA-4926^T^	PZ366123	G	Y	Y	Y	Y	Y	M	S	Y	Y	G	Y	R											
*P. matreminventam* sp. nov.	22FFX-029B^T^	PZ170371	G	T	C	T	T	C	C	G	C	C	G	T	A											

### Taxonomy

***Phytophthora matreminventam*** D. Bily, X. Yang, T. Gyatso, sp. nov.

Fig 3 MycoBank: MB863541 [urn:lsid:mycobank.org: MB863541].

**Fig 3:**
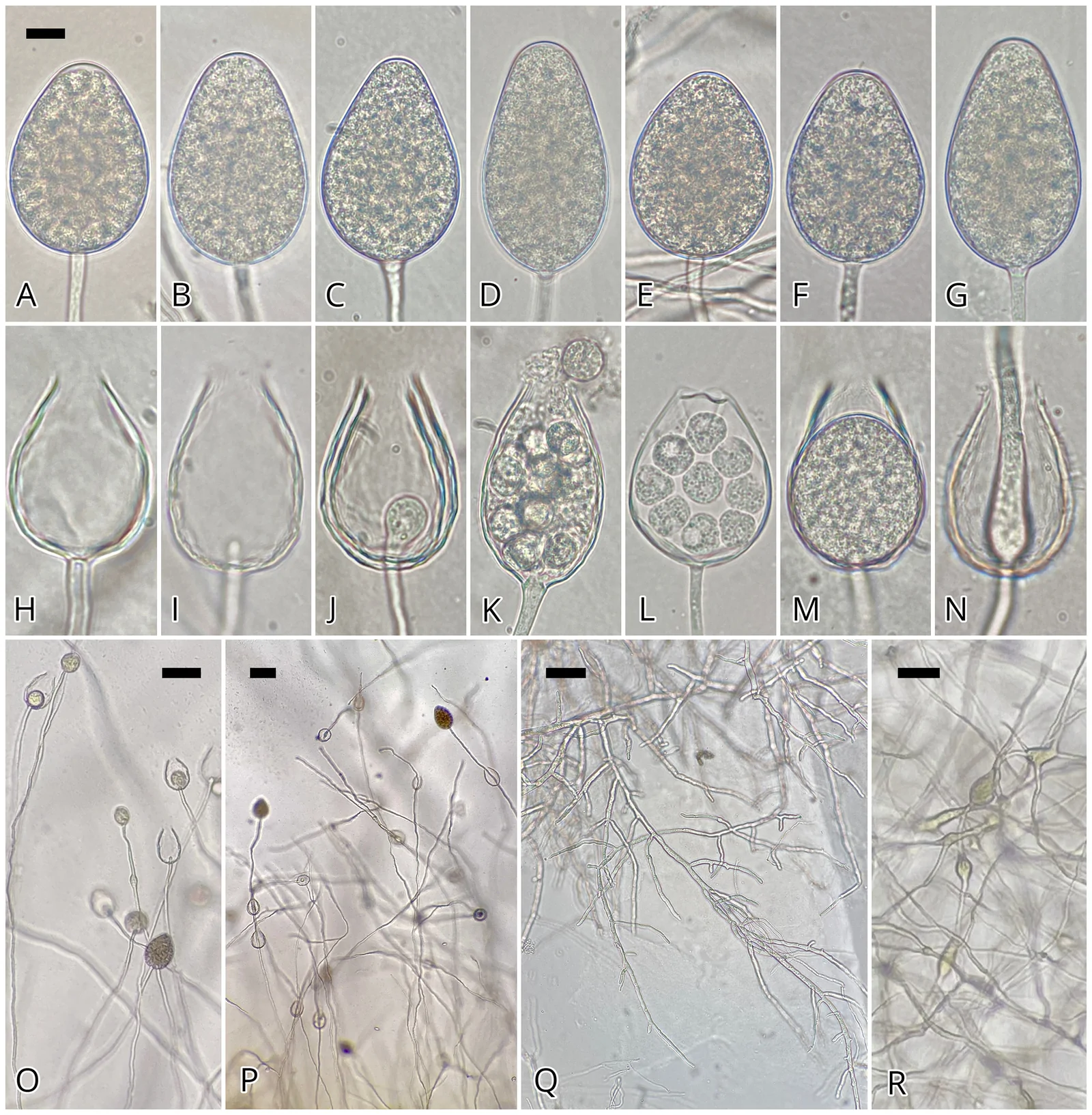
Morphology of *Phytophthora matreminventam* sp. nov. (A–G) Ellipsoid, ovoid, obpyriform, non-papillate sporangia; (H–J) wide sporangial exit pore; (K and L) subglobose zoospores within a sporangium; (M) nested sporangial proliferation (N) internal sporangial proliferation; (O and P) abundant sporangia flooded in spring water for 24 hours with nested and internal proliferation; (Q) hyphae in clarified V8 medium; (R) hyphal swellings 48 hours after flooded in spring water. (A–N) bar = 10 um, (O and P) bar = 30 um, (Q and R) bar = 20 um.

#### Morphological structures on V8A.

Sporangia not formed in solid agar, abundant when flooded in spring water for 24 h. Sporangia borne terminally on long sporangiophores, unbranched, non-papillate with an apical thickening, persistent, ellipsoid, ovoid, to obpyriform. Sporangia measuring 53.4 ± 6.2 × 37.2 ± 3.9 µm (*n* = 40), length to width ratio 1.43. Sporangial proliferation internal and nested, discharged sporangia with an opening 11–16 µm wide. Zoospores limoniform to reniform while motile, spherical on encystment, measuring 10–13 µm in diameter. Oogonia or oospores not observed in single culture. Chlamydospores not observed in solid agar or when flooded in spring water. Hyphae aseptate, hyaline, straight, occasionally sinuous with knobby protrusions, 3.5–8 µm in diameter, hyphal swellings in spring water after 48 h.

#### Holotype.

CBS H-25854 (ex-type CBS 155235, sample collection *22FFX-29B*), isolated from a *Rhododendron maximum* leaf baited in stream water, Fairfax County, Virginia, November 2022. Other isolates collected from stream water include: 21FFX-26, Dec 2021; 21FFX-31B, Dec 2021; 21FFX-35, Dec 2021; 22FFX-27C, Nov 2022; 22FFX-31B, Nov 2022; 22FFX-40A, Nov 2022; 23FFX-12B, May 2023; 23FFX-20C, Oct 2023; 23FFX-28A, Nov 2023; 24FFX-12A, May 2024.

#### Etymology.

Latin translating to “the mother having been found” or “mother found.”

#### Culture characteristics.

V8A: Colonies with dense, aerial, cottony mycelia; no pattern formed. CMA-PARP agar: Colonies forming a petaloid pattern with aerial mycelia in the center that becomes submerged towards the margin. CMA: Colonies faint, submerged, forming a faint petaloid pattern. MEA: Colonies dense, with felty aerial mycelium and fringed, irregular margins. PDA: Colonies with cottony mycelia forming a rosette-like pattern with irregular margins (Fig 4).

**Fig 4:**
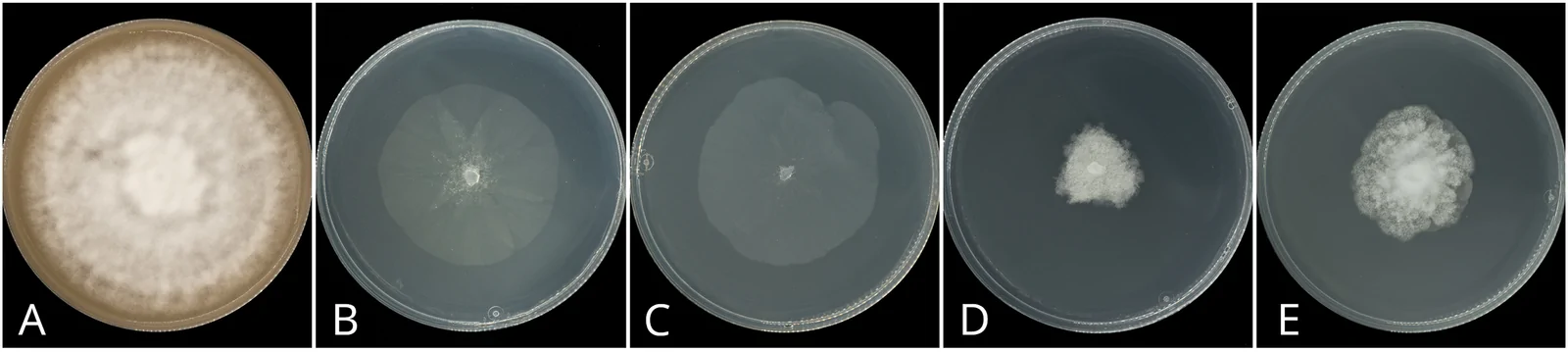
Cultural characteristics of *Phytophthora matreminventam* sp. nov. isolate 22FFX-029B on (A) V8 juice agar, (B) CMA-PARP agar, (C) corn meal agar, (D) malt extract agar, and (E) potato dextrose agar after 7 days at 25°C under ambient light.

#### Cardinal temperatures.

*Phytophthora matreminventam* sp. nov. is a thermophilic species with a minimum growth temperature at 5 °C, optimum at 25 °C, and maximum at 35 °C (Fig 5). Its minimum lethal temperature is 2.5 °C and its maximum is 37.5 °C (Fig 5).

**Fig 5:**
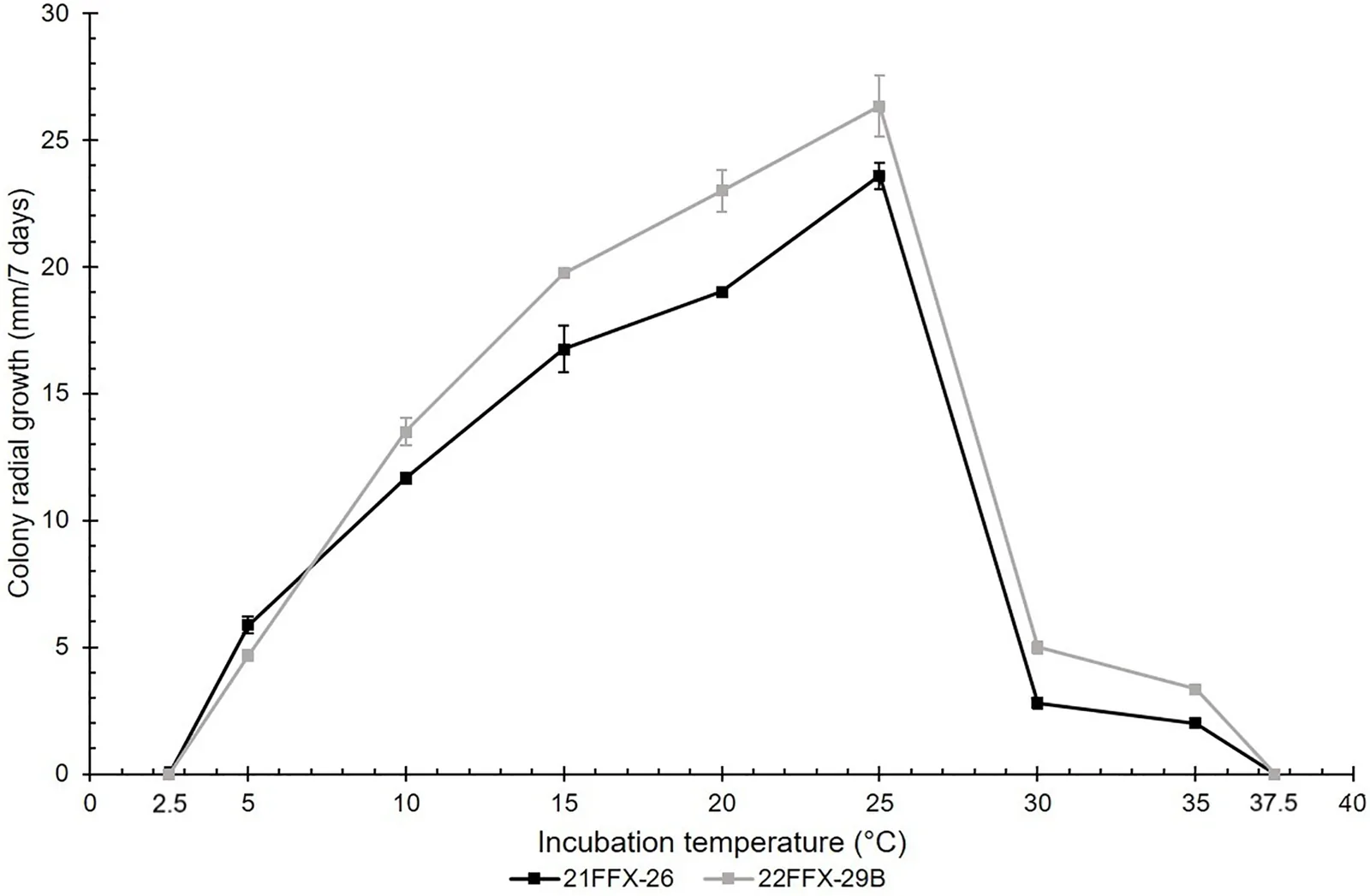
The average radial growth rate of two *Phytophthora matreminventam* sp. nov. isolates at nine incubation temperatures on clarified V8 juice agar after 7 days. Bars represent standard deviation between the three replicates.

#### Notes.

*Phytophthora matreminventam* sp. nov. is placed in a distinct lineage in subclade 6b, ancestral to *P. gonapodyides* and *P. chlamydospora* (Fig 2). Morphologically, it is challenging to distinguish *P*. *matreminventam* sp. nov. from its closest relatives. It differs from *P. chlamydospora* by its larger sporangia that are exclusively borne terminally, and lack of chlamydospores [41]. It differs from *P. gonapodyides* by its wider sporangia without a tapered base [37].

## Discussion

We report a diverse assemblage of *Phytophthora* taxa from forested urban streams of Fairfax County, Virginia, including *Phytophthora matreminventam* sp. nov., the maternal parent of *P*. × *stagnum*. Except for *P. matreminventam* sp. nov., *P*. sp. × Hennops, and *P*. taxon polonicoides, all taxa identified in this study have previously been detected from waterways in the eastern United States [3–5,9,10,16,38,39,41,43,47,48]. Some species previously recovered from nursery irrigation water in Virginia (*P. cactorum*, *P. citrophthora*, *P. cryptogea*, *P. drechsleri*, *P. megasperma*, *P. palmivora*, *P. syringae*, *P. tropicalis*) were not detected in this study, indicating an uncommon occurrence in natural waterways of Fairfax County [55,56]. Based on our survey results, *P. matreminventam* sp. nov. appears to be a common inhabitant in waterways within our sampling area, but the pathogenicity and ecology of the species is unknown.

Species in clade 6 including *P. chlamydospora*, along with clade 9 species including *P. honggalleglyana*, *P. irrigata*, and *P. virginiana* sensu lato were most recovered in this survey (Table 3). These species were also the most abundant taxa in other stream surveys in the United States [3–7,16,]. Most species in clades 6 and 9 are thermophilic and some produce resting structures that can survive extreme environmental conditions, which likely contributes to their wide and variable distribution [2,27,48,57]. For example, *P. chlamydospora* and *P. virginiana* sensu lato both produce abundant chlamydospores and can tolerate temperatures up to 35 °C [41,43]. Interestingly, the abundance of two clade 6 species, *P. gonapodyides* (*n* = 3) and *P. lacustris* (*n* = 3), was much lower compared to results from two stream surveys in western USA, where the two species represented 37% and 47% of all isolates recovered, respectively [8,23].

Some taxa identified in this survey have been detected for the first time in the United States, including *P*. sp. × Hennops, which has previously only been reported from streams and soils of South Africa [49], and *P.* taxon polonicoides, a distinct heterothallic species closely related to *P. polonica* with large chlamydospores and non-papillate sporangia (Fig 2). Some taxa have been reported infrequently, including *P.* taxon bitahaiensis, which has been detected from forest streams in China and Vietnam but only reported in the United States from Pennsylvania [3,44,45]. Finally, *P. pseudocitrophthora* has only been identified in the United States from Louisiana and Massachusetts [38]. The distribution of these species, and whether they are endemic to streams of the eastern United States is unclear.

In this survey, significantly more isolates were recovered in fall (*n* = 119) compared to spring (*n* = 60) (Table 3). Due to the abundance of deciduous tree species, leaf litter in waterways is much higher in the fall compared to spring. Experiments have shown that many aquatic species, including clade 6 species like *P. gonapodyides*, feed on brown, decomposed leaves [2,27]. Ample food, along with increased precipitation were most likely responsible for higher recovery rates in the fall [12].

We used *Rhododendron* leaves to bait our water samples because they are attractive to a wide variety of *Phytophthora* taxa [6,7,58]. We also used the *in situ* ‘bottle of bait’ method instead of the *ex situ* floating leaf bags, which can be placed in the stream for up to one week, increasing the leaves’ exposure to zoospores [59]. Additionally, filtering stream water can increase detection sensitivity and quantify zoospore propagules in a sample [58]. Using multiple bait types, filtering water, and employing mesh bags in the stream for longer periods would most likely have expanded the quantity and diversity of *Phytophthora* taxa [4,59].

The species richness and distribution of *Phytophthora* taxa in the United States have undoubtedly expanded due to influences from urban development, agriculture, and the plant nursery trade [4,15,19,60]. It is possible that *Phytophthora matreminventam* sp. nov. is also present in plant production systems given that *P*. × *stagnum* was first reported from nursery irrigation reservoirs in Virginia [24]. More stream surveys are needed in the eastern United States to better understand the ecology and distribution *Phytophthora* populations in aquatic ecosystems, especially *P. matreminventam* sp. nov.
